# Triptolide Shows High Sensitivity and Low Toxicity Against Acute Myeloid Leukemia Cell Lines Through Inhibiting WSTF-RNAPII Complex

**DOI:** 10.3389/fonc.2022.811850

**Published:** 2022-02-16

**Authors:** Di Kang, Yan Liu, Yi Song, Bingqian Fang, Qichun Zhang, Lihong Hu

**Affiliations:** Jiangsu Key Laboratory for Functional Substance of Chinese Medicine, School of Pharmacy, Nanjing University of Chinese Medicine, Nanjing, China

**Keywords:** triptolide, triptolide-sensitive cell lines, acute myeloid leukemia, antitumor mechanisms, toxicity, WSTF-RPB1

## Abstract

Triptolide exhibits superior and broad-spectrum antitumor activity. However, the narrow safety window caused by the toxicity of triptolide limits its clinical applications. Although several characterized targets for triptolide are reported, the association between triptolide and its targets in cancer therapy is not fully understood. Here, we show that acute myeloid leukemia (AML) cell lines are sensitive to triptolide by constructing an *in vitro* cell and *in vivo* xenograft models. Meanwhile, the triptolide-induced hepatotoxicity increases with increasing dosages within the xenograft models. Additionally, the expression levels of WSTF-RPB1 are strongly associated with the sensitivity to triptolide in hematological cancer cells and can be downregulated in a dose and time-dependent manner. Finally, we show that optimizing dosing regimens can achieve the same pharmaceutical effect and reduce toxicity. In summary, this study aims to search for triptolide-sensitive cell lines as well as the underlying molecular mechanisms in order to broaden the safety window of triptolide; thus, increasing its clinical utility.

## Introduction

Triptolide is a natural compound extracted from the Chinese medical herb *Tripterygium wilfordii* Hook F and possesses a wide range of pharmacological effects, including anti-inflammatory, immunosuppressive, and anticancer activities (1). In the field of oncology, triptolide exerts potent antitumor activities in a variety of cancers, including hematological tumors such as AML and myeloma, as well as solid tumors such as pancreas and breast cancer (2). However, the narrow therapeutic window caused by severe systemic toxicity limits its clinical application (3–6). Some preclinical studies have reported that the IC_50_ values of triptolide against cancer cell lines vary (7–12). The discovery of sensitive cell lines to triptolide may provide a possibility to broaden its safety window and its clinical application.

Several targets and signaling pathways are reported for triptolide in cancer therapy. Triptolide affects cell proliferation, cell apoptosis, cell cycle, cancer stem cells, and the tumor microenvironment (1, 2, 12). In particular, the covalent binding of triptolide to Xeroderma pigmentosum group B (XPB), one subunit of the TFIIH transcription complex regulating RNA polymerase II (RNAPII), makes triptolide a unique transcriptional inhibitor that downregulates many proteins involved in distinct signaling pathways (13). In addition to XPB, TGF-beta-activated kinase-1 binding protein 1 (TAB1) in macrophages and other proteins have been reported to interact with triptolide (14). However, little is known about the interaction of triptolide with these targets and their relationships in cancer therapy; thus, helping to contribute to the understanding of the broad anticancer activity of triptolide. As an irreversible inhibitor *via* covalent binding to Cys342 of XPB, triptolide shows potent antiproliferative activity in HEK293T cells. The engineered XPB-C342 mutant cell line conferred resistance to triptolide, validating XPB as a target in HEK293T cells (15). Whereas the proteasome-dependent degradation of the largest subunit of RNA polymerase II (RPB1), not XPB, contributes to the cell killing activity of triptolide, providing an insight to other targets and mechanisms that may exist for triptolide in cancer therapy (16).

Numerous studies have indicated that chemo and targeted therapies induce DNA damage and genome instability through double-strand breaks (DSBs). Accumulation of DNA damage in cells promotes enhanced DNA repair activities, protecting the cell against DNA damage sustaining genome stability (17). The inhibition of this mechanism of action allows for the eradication of actively proliferating cells by targeting the DNA repair signaling pathways. In recent years, triptolide was reported to induce DNA damage and is sensitive to chemotherapy in murine B-cell lymphoma cells (9), human melanoma (18), laryngocarcinoma (19), and breast cells (20). The largest subunit of RNAPII (RPB1) is responsible for AML cell overgrowth and plays an important role in the process of the DNA-damage response (21, 22). One study reported that RPB1 maintains genome integrity through the Williams syndrome transcription factor (WSTF) interaction. The chromatin remodeler WSTF has an atypical tyrosine kinase activity. The translocation of RPB1-WSTF to DNA lesions facilitates DNA repair by homologous recombination in the G1 cell cycle phase (23).

In this study, we tested the sensitivity of triptolide to 15 selected cancer cell lines ranging from hematological to solid tumor cells *in vitro* and *in vivo*. We reported that low-lose triptolide showed potent anticancer activity against AML cells *in vitro* cell models and in *in vivo* xenograft models. Additionally, triptolide induced apoptotic cell death and cell cycle arrest at G1 phase. Moreover, we observed the toxicity of triptolide in tumor-bearing nude mice at different doses. Then, we studied the interaction between the enhanced anti-AML activity and the different molecular targets. AML is a cancer of the blood and bone marrow, and AML patients response to tyrosine kinase inhibitors initially, but develop to resistance in a short duration time (24–28). In summary, our data suggest that triptolide is a potential anti-AML agent, and we are the first to report that the sensitivity of AML cells to triptolide is strongly associated with the expression of WSTF-RPB1.

## Materials and Methods

### Chemicals and Reagents

Triptolide (#ST02050120, C_20_H_24_O_6_, MW: 360.4, ≥98% purity) was purchased from Shanghai Standard Technology Co., Ltd. (Shanghai, China); Dimethyl Sulfoxide (#G75927B, (CH_3_)_2_SO, MW: 78.13, ≥99% purity) was purchased from Shanghai Titan Scientific Co., Ltd. (Shanghai, China); 4x Laemmli Sample Buffer (#1610747) was purchased from Bio-Rad Laboratories, Inc. (Hercules, CA); Olive oil (#0815211) was purchased from Shanghai Macklin Biochemical Co., Ltd. (Shanghai, China). Trypsin (#25200072) was purchased from Thermofisher Scientific Co*.*, Ltd. (Shanghai, China).

### Cell Culture

The human cancer cell lines, including HepG2 (#TCHu 72), HT-29 (#TCHu103), Hep3B (#SCSP-5045), Hela (#TCHu187), A549 (#TCHu150), were purchased from the Cell Bank of Chinese Academy of Sciences (Shanghai, China). HepG2, Hep3B, Hela, A549, and MDA-MB-231 (#HTB-26, ATCC) were grown in high-glucose Dulbecco’s Modified Eagle Medium (DMEM, #C11995500BT, Gibco) with 10% fetal bovine serum (#10091148, Gibco) and 1% penicillin–streptomycin (#15140122, Gibco). HL-60, U937, CAG, ARP-1, and H929 were kind gifts from the Jiangsu Provincial Hospital. The above five cell lines and THP-1 (#TIB-202, ATCC), HT-29 (#TCHu103), HCT116 (#CCL-247, ATCC) were cultured in RPMI 1640 medium (#C11875500BT, Gibco), and MV-4-11 (#CRL-9591, ATCC) and KG-1 (#CCL-246, ATCC) were grown in Iscove’s Modified Dulbecco Medium (#30-2005, ATCC) supplemented with 10% FBS and 1% penicillin–streptomycin.

### Cell Viability Assay

The viability of the leukemia cells (MV-4-11, KG-1, THP-1, HL-60), lymphoma cells (U937) and myeloma cells (CAG, ARP-1, H929) was assessed using the Cell Counting Kit-8 (#CK04, DoJINDO). The cells were seeded into 96-well plates, and treated with triptolide at various concentrations for 48 h. Then the viable cells were stained with 10 μL of CCK-8 solution for another 2 h. Following incubation, the absorbance was measured at 450 nm using a Microplate Reader (Infinite M Nano, Nanjing Cleande Scientific Instrument Co., Ltd.). 3-(4,5-dimethylthiazol-2-yl)-2,5-di-phenyltetrazolium bromide (MTT, #HY-15924, MCE) reagent was used for evaluating the viability of solid tumor cells. All adherent cells were in the logarithmic growth phase, including the HepG2, HT-29, MDA-MB-231, Hep3B, HCT116, Hela, and A549 cells. All the cell lines were seeded into 96-well plates for 12 h. After attachment, they were treated with triptolide for 48 h. Afterward, viable cells were stained with 20 μL of MTT solution (5 mg/mL) for another 4 h. Finally, the supernatant was removed, and the absorbance of the formazan crystal, dissolved in 150 μL DMSO, was measured at 490 nm using a Microplate Reader (Infinite M Nano, Nanjing Cleande Scientific Instrument Co., Ltd).

### Cell Apoptosis Assay

2×10^6^ cells/well MV-4-11 and THP-1 cells were seeded into 6-well plates and were treated with triptolide at various concentrations for 48 h. Following the incubation, the cells were rinsed three times with cold PBS (#P1020, Solarbio) and stained with Annexin V-FITC/PI kit (#A211-02, Vazyme) for 15 min at room temperature. Finally, cell apoptosis analysis was performed using a Gallios (BECKMAN COULTER) and the Kaluza C Software.

### Cell Cycle Arrest Assay

MV-4-11 and THP-1 cells in six-well plates at a density of 2×10^6^ cells/well were exposed to triptolide at different dosage for 48 h. After treatment, the cells were harvested and rinsed three times with cold PBS. Then cells were fixed with 70% ice-cold ethanol at 4°C overnight. On the second day, the cells were washed with cold PBS three times, then stained with PI/RNase staining buffer (#550825, BD Pharmingen) for 30 min. The cells were evaluated and analyzed by Gallios (BECKMAN COULTER). Lastly, the cell cycle distribution at different phases was analyzed using Kaluza C Software.

### Western Blot Assay

MV-4-11 and THP-1 cells were treated with triptolide at various concentrations (5, 10, and 20 nmol/L) for 6 h, or treated with triptolide (20 nmol/L) for 30, 60, 90, 120, 240, and 360 min. Then the cells were washed twice with cold PBS and then lysed on ice for 30 min using RIPA lysis buffer (#20101ES60, Beyotime) with the addition of Nuclease (#20156ES50, YEASEN) and Pierce™ Protease and Phosphatase Inhibitor Mini Tablets, EDTA-free (#A32961, ThermoFisher). Finally, the cells were centrifuged at 12,000 g for 15 min and then the supernatant was collected. For Western blot analysis, Precision Plus Protein™ Dual Color Standards (#1610747, BIO-RAD) and protein samples were separated by SDS-PAGE at a constant voltage of 120 V for 1.5 h and transferred to a PVDF membrane at 100 V. After blocking with 5% non-fat milk for 2 h at room temperature, PVDF membranes were incubated with the specific primary antibody at 4°C overnight. Next day, PVDF membranes were incubated with the secondary antibody for 2 h at room temperature before blocking for 1 h with 5% skim milk, and finally were washed three times with TBST. Subsequently, the signal was detected with SuperSignal™ West Femto Maximum Sensitivity Substrate (#34096, ThermoFisher) using a Tanon 5200. The primary antibodies used were mouse anti-RPB1 (#2629, CST), rabbit anti-Phospho-RPB1 (#4735, CST), mouse anti-WSTF (#514287, Santa Cruz), mouse anti-WSTF (#2152, CST), mouse anti-XPB (#271500, Santa Cruz), mouse anti-XPB (#8746, CST), rabbit anti-TAB1 (#3226, CST), rabbit anti-Phospho-P38 (#4511, CST), rabbit anti-P38 (#8690, CST), rabbit anti-Phospho-Histone H2A.X (Ser139) (#2577, CST), rabbit anti-Phospho-Histone H2A.X (Tyr142) (#12101, SAB), rabbit anti-Histone H2A.X (#7631, CST), and mouse anti-β-ACTIN (#21338, SAB). The secondary antibodies were Peroxidase AffiniPure Goat Anti-Rabbit IgG (H+L) (1:10000, Jackson ImmunoResearch) and Peroxidase AffiniPure Goat Anti-Mouse Rabbit IgG (H+L) (1:5000, Jackson ImmunoResearch).

### *In Vivo* Xenograft Growth Study

Male and female BALB/c Nude mice (4 weeks old) were purchased from GemPharmatech Co., Ltd. (Nanjing, China). They were housed under a pathogen-free condition with a temperature maintained between 20-26°C and a relative humidity of 40%-70%. The protocol was performed and approved by the Animal Ethics Committee of Nanjing University of Chinese Medicine (Nanjing, China). Tumor cells were collected during the logarithmic phase of growth and washed twice with PBS prior to implantation. MV-4-11, THP-1 human leukemia cells, or MDA-MB-231 breast cancer cells (5×10^6^ cells/mouse) in PBS were suspended as a 1:1 mixture in Matrigel (#356234, Corning) and subcutaneously injected into the right flank of each nude mouse. Once the tumors reached 100-300 mm^3^ in size, the mice were randomly divided into the control or treatment groups, and received daily intraperitoneal injection of solvent control or triptolide. Bodyweight and tumor volume were measured daily. The tumor volume of the mice was calculated using the formula: Tumor Volume (mm^3^) =length (mm) × [width (mm)]^2^×0.5.

### Hematoxylin & Eosin Staining

At the end of the experiment, the mice were sacrificed. Tumor xenografts and organ tissues were removed from the mice, and fixed properly by 4% paraformaldehyde fix solution (#C104188, Aladdin) prior to sectioning. After fixation, the paraffin-embedded tissues were cut into 4-6 μm thickness, which were subsequently placed on positively charged slides. The slides were then deparaffinized and rehydrated to remove with paraffin xylene and Ethanol. The rehydrated tissue sections were dipped into a Coplin jar containing hematoxylin solution (#G1004, Servicebio) for 30 sec and rinsed in running tap water for 1 min. Subsequently, the slide was stained with 1% eosin Y solution (#G1001, Servicebio) for 10-30 sec, and then dehydrated with 95% alcohol twice and 100% alcohol (#100092683, *Sinopharm Chemical Reagent* Co., Ltd.) twice for 30 sec. Next, two changes of xylene were used for extracting the alcohol in the slides. Finally, an appropriate mounting medium was added and the slides were covered with a coverslip.

### Immunohistochemistry Staining

After fixation, deparaffinization, and rehydration as the same performed in 2.7, heat-induced antigen retrieval was carried out in 10 mM sodium citrate buffer (PH=6.0) (#G1202, Servicebio), and occurred in a microwave oven for 15 min. Next, the slides were washed in TBST twice and blocked in TBS with 5% goat serum for 1 hour at room temperature. Then, they were incubated with the primary antibodies of Ki67 (#GB111141, 1:1000, Servicebio) and Caspase-3 (#GB11009-1, 1:300, Servicebio) at 4°C overnight. Next day, the TBST buffer were used for washing the slides at least three times before blocking in TBS buffer with 5% goat serum for 1 hour. Then, the secondary antibody HRP conjugated Goat Anti-Rabbit IgG (H+L) (#GB23303, 1:200, Servicebio) was added for antibody detection. Finally, the slides were washed twice with TBST and mounted with a mounting solution.

### Ethics Statement

All animal experiments were performed and approved by the ethics committee of Nanjing University of Chinese Medicine.

### Statistical Analysis

Graphpad Prism 8.0 software was used to analyze all data for statistically significance. The data were represented as mean ± SD of at least three independent experiments. Statistical significance was assessed by using the Student’s *t-*test between the two groups.

## Results

### Leukemia Cell Lines Are Significantly More Sensitive to Triptolide *In Vitro*

**Figure 1:**
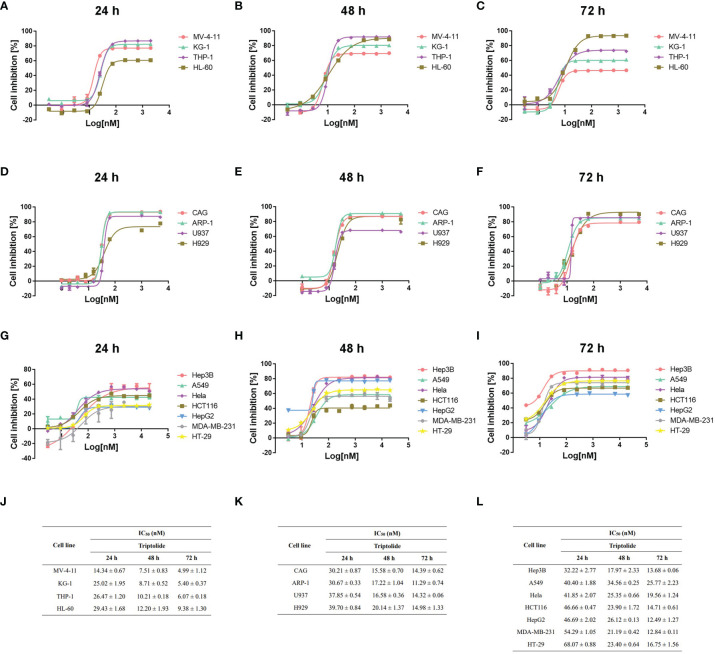
Triptolide induces cytotoxic effects on different cell lines *in vitro*. **(A–I)** Fifteen different cell lines, including AML **(A–C)**, other hematological cell lines **(D–F)** and solid cancer cell lines **(G–I)** were exposed to triptolide for 24 hours **(A, D, G)**, 48 hours **(B, E, H)** and 72 hours **(C, F, I)**, respectively. Cell viability was analyzed using cellular cytotoxicity assays. **(J–L)** IC_50_ values in different cell lines for triptolide are shown. The data is expressed as the mean ± SD based on three independent experiments.

To clarify the antineoplastic activity for triptolide, we screened 15 cancer cell lines derived from different cancer cell types ranging from hematological to solid tumors. Different cancer cell lines were exposed to increasing concentrations of triptolide for 24, 48, or 72 hours. And the half-maximal inhibitory concentration (IC_50_) was calculated using cellular cytotoxicity assays. After treatment with triptolide for 24 hours, all leukemia cell lines including MV-4-11, KG-1, THP-1 and HL-60 showed sensitivity with IC_50_ concentrations below 30 nM/L (**Figures 1A, J** **)**, whereas other cancer cell lines were less sensitive to triptolide with higher IC_50_ above 30 nM/L (**Figures 1D, G, K, L** **)**. To further evaluate the cellular cytotoxicity of triptolide, we tested the IC_50_ of triptolide in all cancer cell lines for 48 (**Figure 1B, E, H**) and 72 hours (**Figure 1C, F, I**). As shown in **Figure 1**, triptolide showed time-dependent cytotoxicity in leukemia cells, with the IC_50_ decreasing over time (**Figure 1J**). The IC_50_ of triptolide in leukemia cells were below 15 nM/L and 10 nM/L after treatment for 48 and 72 hours, compared with other cancer cells (**Figures 1E–I, K, L** **)**. In summary, cell viability experiments *in vitro* indicate that triptolide is more sensitive in leukemia cell lines compared to other hematological or solid tumor cell lines.

### Triptolide Induces Apoptotic Cell Death and Cell Cycle Arrest in Leukemia Cell Lines

**Figure 2:**
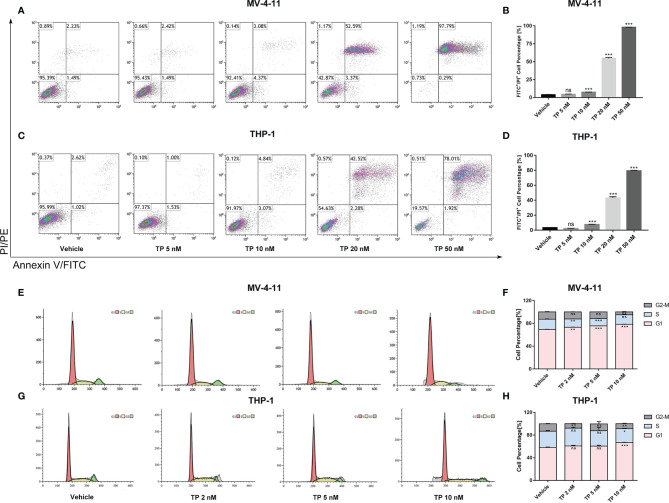
Triptolide induces cell apoptosis and cell cycle arrest in MV-4-11 and THP-1 cells. **(A–D)** Treatment with triptolide at various concentrations in MV-4-11 **(A, B)** or THP-1 **(C, D)**. After 48 hours, apoptotic cells were stained with dual Annexin-FITC/PI staining and examined by flow cytometry. **(B, D)** Bar graphs represent the percentage of cells at early and late stages of cell apoptosis. **(E–H)** Representative flow cytometric histograms, showing cell-cycle distribution in MV-4-11 **(E, F)** and THP-1 **(G, H)**, were presented after triptolide treatment for 48 hours. **(F, H)** Bar graphs represented the percentage of cells in different cell cycle phases. Data are shown as the mean ± SD from three independent experiments. *p < 0.05; **p < 0.01; ***p < 0.001. ns, no significance.

Cell apoptosis assay using Annexin V/PI staining was performed and analyzed *via* flow cytometry to determine whether triptolide affects apoptotic cell death. Triptolide treatment for 48 h with increasing concentration resulted in cellular apoptosis of both MV-4-11 and THP-1 in a dose-dependent manner. Triptolide treatment at the doses of 5, 10, 20, and 50 nM/L induced 4.44%, 7.56%, 54.70% and 98.07% cell apoptotic death in MV-4-11 cells on average, respectively (**Figures 2A, B** **)**. The total apoptotic death accounted for 2.37%, 7.52%, 43.02%, and 79.38% on average in THP-1 cells at the doses of 5, 10, 20, and 50 nM/L (**Figures 2C, D** **)**. Thus, triptolide can induce apoptotic cell death in leukemia cell lines. Additionally, we further examined the effects of triptolide on cell cycle progression for both cell lines. The flow cytometry showed that triptolide induced cell cycle arrest at the G1 phase, resulting in increased G1 phase-cell population at the expense of a decrease in the proportion of cells in other phases (**Figures 2E–H**). For MV-4-11, the G1 phase-cell population increased from 69.40% to 73.11%, 75.55%, and 78.55% when treated with triptolide at doses of 2, 5, 10 nM/L for 48 hours, respectively (**Figures 2E, F** **)**. The cell cycle in THP-1 cells treated with triptolide at the same dosage as that in MV-4-11 was also performed, showing the increase of G1 phase cell population from 58.13% to 60.75%, 60.66%, and 66.85% (**Figures 2G, H** **)**. In summary, triptolide induces apoptotic cell death and impedes cell cycle progression in leukemia cell lines.

### Low-Dose Triptolide Shows Potent Anti-Leukemia Effect *In Vivo*

**Figure 3:**
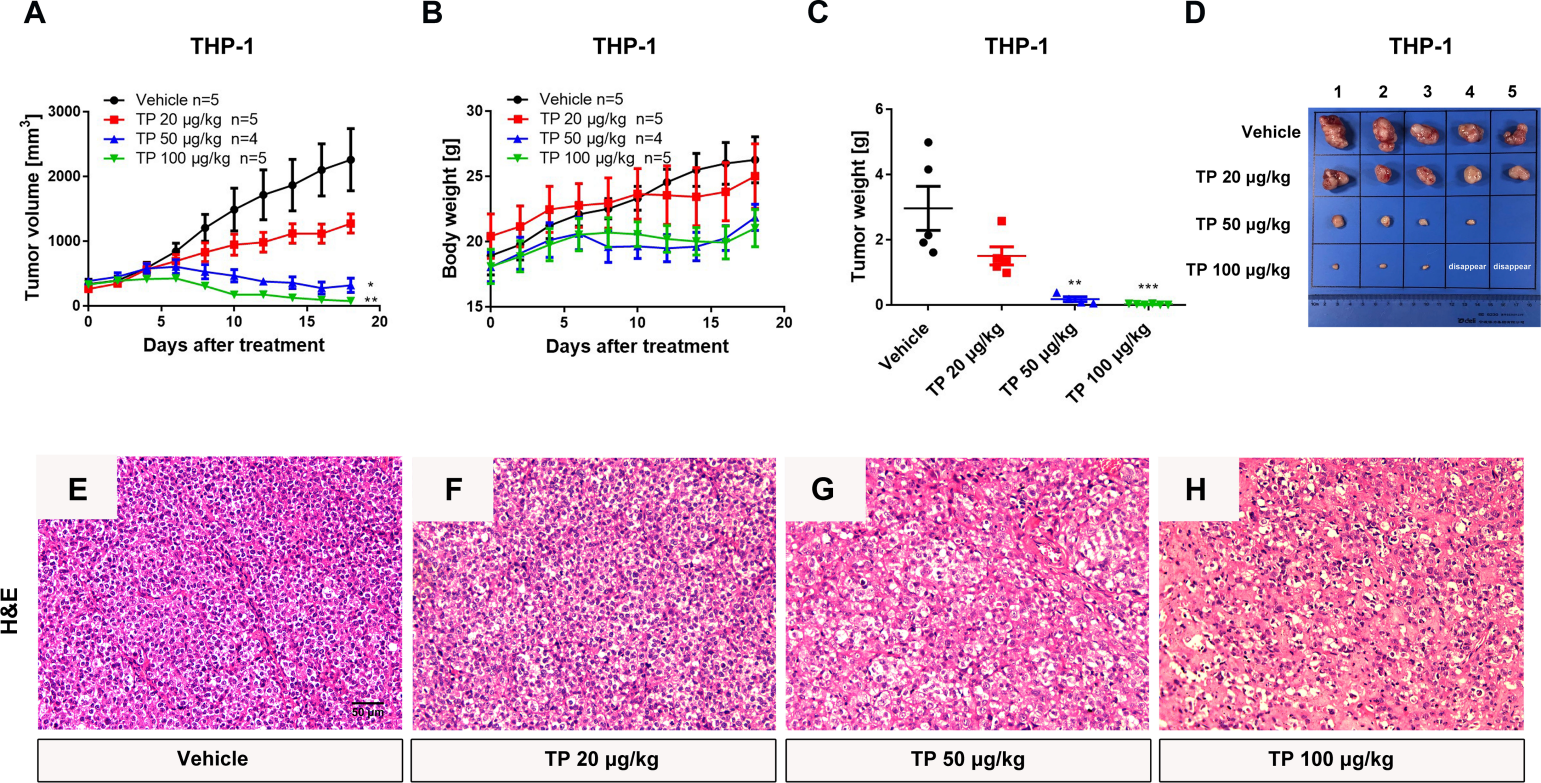
Low-dose triptolide reveals the anti-AML activity in a THP-1 xenograft model. 5×10^6^ THP-1 cells were injected into BALB/C nude mice subcutaneously. When the tumor volume reached 100-200 mm^3^, mice were randomly assigned to four treatment groups and treated with triptolide at a dosage of 20, 50, and 100 μg/kg/day *via* an intraperitoneal injection for 18 consecutive days. Tumor volume **(A)** and bodyweight **(B)** was measured every other day. At the end of the study, tumors were weighed **(C)** and photographed **(D)**. *p < 0.05; **p < 0.01; ***p < 0.001; **(E–H)** Representative photos of tumor sections stained with H&E were represented.

**Figure 4:**
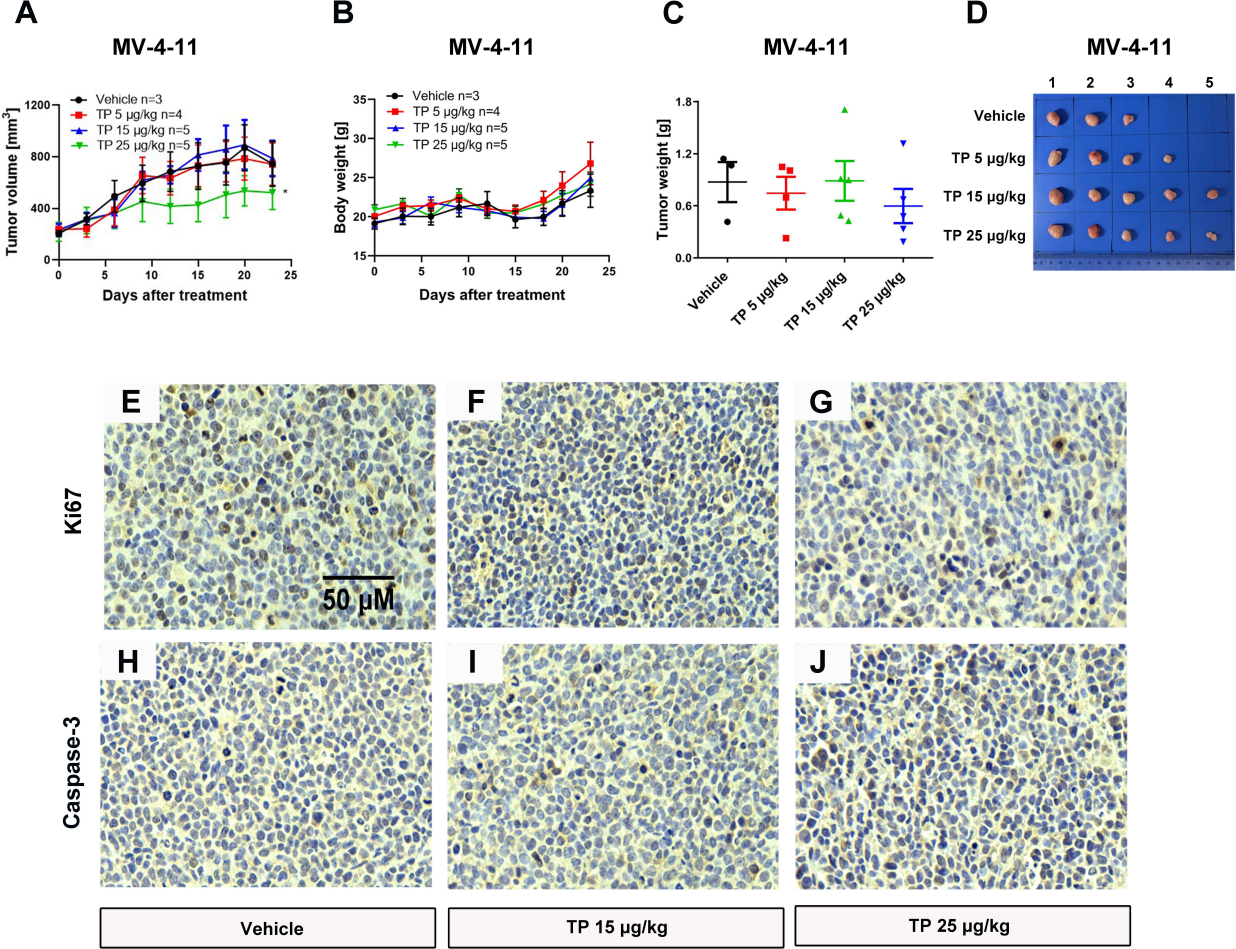
Low-dose triptolide shows a mild anti-AML activity in a MV-4-11 xenograft. 5×10^6^ MV-4-11 cells were injected into BALB/C nude mice subcutaneously. When the tumor volume reached 200 mm^3^, mice were randomly assigned to four treatment groups and treated with triptolide of 5, 15, and 25 μg/kg/day *via* an intraperitoneal injection for 24 consecutive days. Tumor volume **(A)** and bodyweight **(B)** was measured every two days. At the end of the study, tumors were weighed **(C)** and photographed **(D)**. *p < 0.05. Representative photos of tumor sections stained with Ki67 **(E–G)** and Caspase-3 **(H–J)** were represented.

To verify the sensitivity of leukemia cell lines to triptolide *in vivo*, we used nude mice xenograft models to evaluate the antineoplastic effect of triptolide by subcutaneous injection with leukemia or solid tumor cells. In the THP-1 xenograft mouse model, triptolide significantly inhibited tumor growth in a dose-dependent manner, with tumor growth inhibition (TGI) of 49.34%, 94.20%, and 99.36% at dosages of 20, 50, and 100 µg/kg/day for 18 consecutive days, respectively (**Figures 3A–D**). HE staining of the tumor sections showed a marked decrease in cancer cell viability as the dose increased (**Figures 3E–H**). We further validated the antitumor effect against the MV-4-11 xenograft mouse model with low-dose triptolide. Compared to the vehicle control and the lower-dose group, tumor volume and weight were mildly inhibited at the dosage of 25 μg/kg/day administered for 24 consecutive days, with no effect on body weight (**Figures 4A–D**). Additionally, we performed Ki67 and Caspase-3 staining to confirm the effect of triptolide on cell proliferation and apoptosis *in vivo*. The results of the Ki67 immunohistochemistry staining demonstrated that triptolide inhibited MV-4-11 cell proliferation, specifically at the dosage of 25 μg/kg/day (**Figures 4E–G**). Caspase-3 staining showed a higher proportion of apoptotic cells compared to the vehicle control, especially in the higher-dose treatment group (**Figures 4H–J**). In summary, *in vivo* studies indicated that the minimum effective dose (MED) of triptolide for anti-leukemia effect was 25 μg/kg/day.

In the MDA-MB-231 xenograft model, triptolide inhibited tumor growth and decreased tumor weight in a dose-dependent manner for 22 consecutive days, with the TGI of 45.06% and 96.52%, at the dosages of 100 and 500 μg/kg/day, respectively (**Figures S1A–D**). Furthermore, the tumor tissue staining using H&E, Ki67, and caspase-3 demonstrated that triptolide inhibited cell proliferation and induced cellular apoptosis (**Figures S1E–M**). In brief, triptolide exerted a more potent antitumor effect on leukemia cells, compared with solid tumor cells.

### The Toxic Effect of Triptolide in Xenograft Models

**Figure 5:**
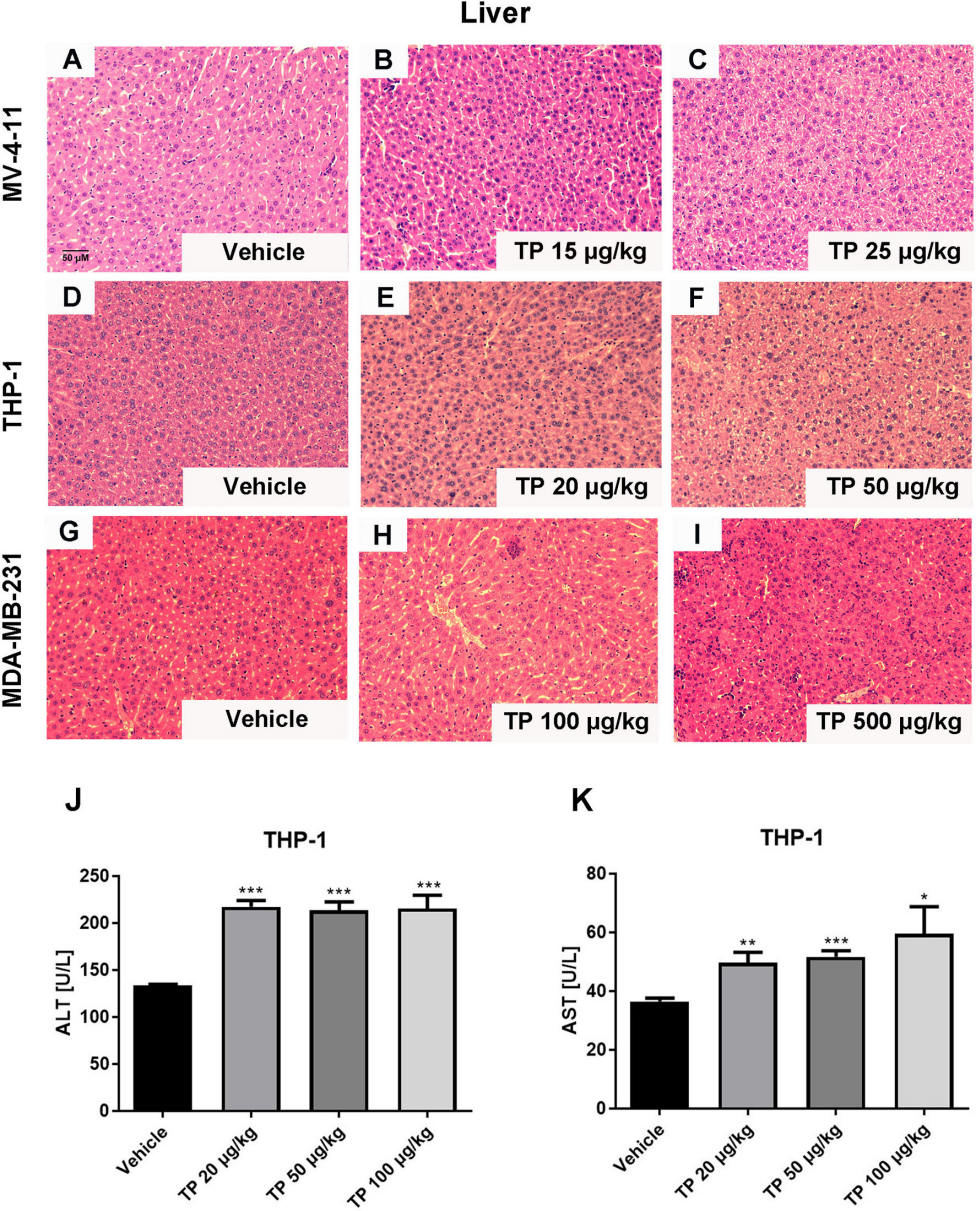
Histopathological changes in the liver after administration with triptolide in different xenograft models. Representative microphotographs of liver sections were stained with H&E (200×) in MV-4-11 bearing mice **(A–C)**, THP-1 bearing mice **(D–F)** and MDA-MD-231 bearing mice **(G–I)**. **(J, K)** ALT and AST levels in the peripheral blood were measured after mice were sacrificed. *p < 0.05; **p < 0.01; ***p < 0.001.

The toxic effect associated with the administration of triptolide by intraperitoneal injection was evaluated at several levels. First, we monitored the death rates and bodyweights upon triptolide administration. No animals died and no serious decrease in body weight was observed within the different dose treatment groups in either the MV-4-11 or THP-1 xenograft animals due to the low-dose administration of triptolide. However, triptolide administration at the dosage of 500 μg/kg/day caused one mouse out of four to die on day 20 in the MDA-MB-231 xenograft model group, and two out of four died in the HepG2 xenograft model group on days 11 and 19 (data not shown). In the THP-1 bearing mice, there was a mild decrease with no statistical significance in bodyweight upon triptolide administration at the dosages of 50 and 100 μg/kg/day (**Figure 3B**). Moreover, toxicity was assessed at the biochemical and histopathology levels. Hepatotoxicity was assessed *via* histopathology of the liver for all groups. Microscopically, the control and low-dose groups in the MV-4-11 xenograft mice showed no remarkable pathological changes in the liver. Whereas, after the administration of triptolide at the dosage of 25 μg/kg/day for 12 days, the H&E staining of the liver sections showed little liver cell degeneration (**Figures 5A–C**). The mild degenerated liver cells were also observed in the THP-1 xenograft mice at the dosage of 50 μg/kg/day (**Figures 5D–F**). Moderate toxicity was observed at the dosage of 100 μg/kg/day for 22 consecutive days, leading to inflammatory cell infiltration and vascular congestion in the liver of the MDA-MB-231 xenograft mice (**Figure 5H**) compared to that of control groups (**Figure 5G**). Furthermore, the highest dosage of triptolide at 500 μg/kg/day induced more severe toxicity in the liver as well as a few hepatocytes that presented with necrotic debris (**Figure 5I**). We also examined alanine transaminase (ALT) and aspartate transaminase (AST) levels in the serum of the THP-1 xenograft mice. The ALT level was significantly increased in all groups, which was 1.6-fold greater (217.12 ± 15.21 U/L, 213.18 ± 18.49 U/L, and 215.26 ± 31.78 U/L) than the vehicle control (133.24 ± 3.05 U/L) (**Figure 5J**). At the same time, the AST levels in the control mice were 36.46 ± 2.51 U/L. Mice treated with increasing doses of triptolide showed a gradual elevation of AST (49.66 ± 7.83 U/L, 51.63 ± 4.10 U/L, and 59.42 ± 20.80 U/L) in plasma (**Figure 5K**). In summary, low-doses of triptolide in tumor-bearing nude mice induced slight liver toxicity, involving increased AST and ALT levels as well as pathological changes. The higher dose of triptolide (500 μg/kg/day) administered for 12 consecutive days led to severe liver degeneration and the death of mice. In comparison with solid tumors, the therapeutic window of triptolide in the treatment of AML was increased approximately 5 times.

### Enhanced Antitumor Activity of Triptolide in AML Xenograft Mice Is Associated With the Downregulation of the RNAPII Complex

**Figure 6:**
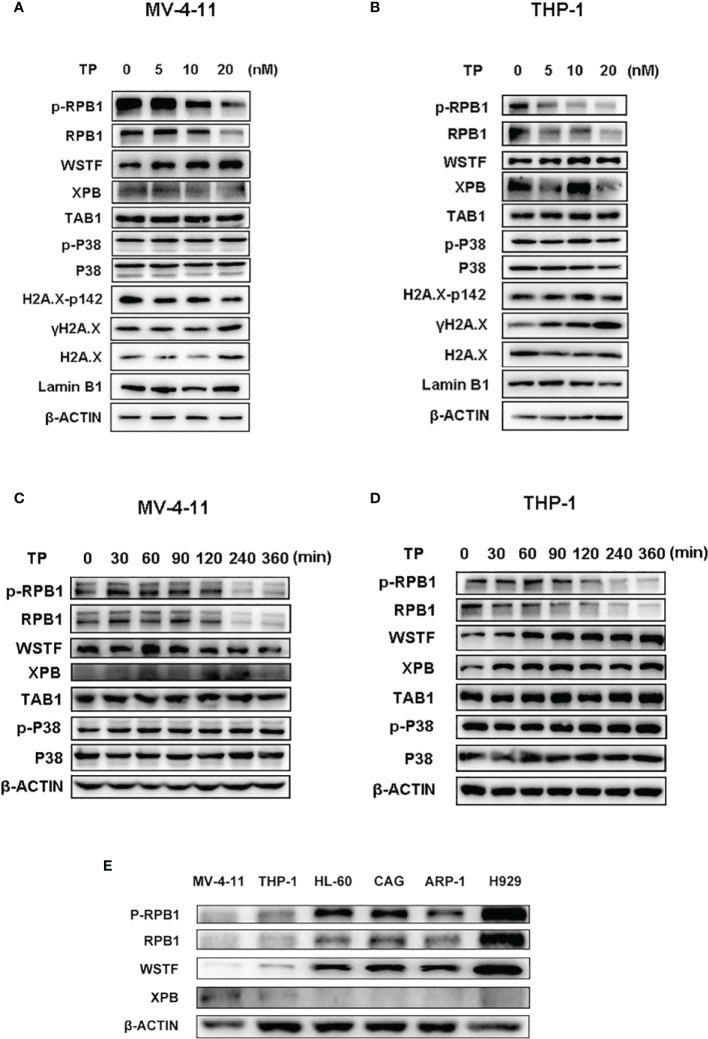
WSTF-RPB1 is strongly associated with the anti-AML activity. Triptolide downregulates the WSTF and RPB1 signaling pathway without affecting TAB1/P38 signaling in a dose dependent manner in MV-4-11 **(A)** and THP-1 **(B)**. Time-dependent experiments show that triptolide inhibits the WSTF-RPB1 signaling pathway with no effect on TAB1/P38 signaling in MV-4-11 **(C)** and THP-1 **(D)**. **(E)** The different expression levels of WSTF, XPB, and RPB1 is shown.

The protein levels of the downstream targets were examined in order to elucidate the mechanism of action of the antitumor activity of triptolide in AML. Triptolide is considered a transcriptional inhibitor, inhibiting the largest subunit of the RNAPII complex (29). Although XPB is one of the most characterized targets of triptolide as a covalent inhibitor, other targets might exist that play essential roles in exerting pharmaceutical effects in different disease states (2, 16). It was reported that downregulation of RNAPII blocks the DNA damage response induced by UV resulting in cell death (18). Furthermore, triptolide was demonstrated as having enhanced chemotherapeutic effect *via* inhibition of the DNA damage response (20). Here, we first tested whether triptolide inhibited the DNA damage response to observe the phosphorylated residue’s expression levels at Ser 139 of H2A.X (λH2A.X), which marks the DNA damage. According to the IC_50_ data in the cellular cytotoxicity assays, we selected triptolide at the dose of 5, 10, 20 nM/L to incubate with cells for 6 hours without affecting much cell apoptotic death. As shown in **Figure 6**, triptolide increased the expression of the DNA damage marker λH2A.X dose-dependently. Simultaneously, it decreased the protein levels of RPB1 and p-RPB1, whereas no change in the TAB1 protein level or the p-P38 expression level was observed, which is downregulated signaling for TAB1. The data indicates that in AML cell lines, triptolide exerts an inhibitory effect on RPB1 blocking the DNA damage response; thus, leading to increases in DNA damage and cell death. In addition, WSTF phosphorylates the tyrosine residue at position 142 (Tyr 142) of H2A.X (pTyr142) and interacts with RPB1 to regulate the DNA repair system. Here we show that triptolide inhibits H2A.X-pY142, resulting in the DNA damage repair defect (**Figures 6A, B** **)**. Furthermore, we performed time-dependent experiments for short time intervals, because triptolide was reported to affect short-lived mRNA. The results showed that the activity of RPB1 in cells was downregulated upon treatment with triptolide and without affecting TAB1 and p-P38 (**Figures 6C, D** **)**. Finally, we observed different expression levels of WSTF and RPB1 in hematological cell lines. The different expression levels of these proteins are strongly associated with the anticancer activity of triptolide. Thus, higher expression levels of WSTF and RPB1 weaken the anticancer activity within the cell lines (**Figure 6E**). In summary, we revealed that WSTF-RPB1 is strongly associated with the anti-AML activity of triptolide, causing diverse sensitivity to triptolide in distinct cancer cell lines.

### The Effect of Changes in the Administration Cycle for Triptolide on Pharmaceutical Activity

**Figure 7:**
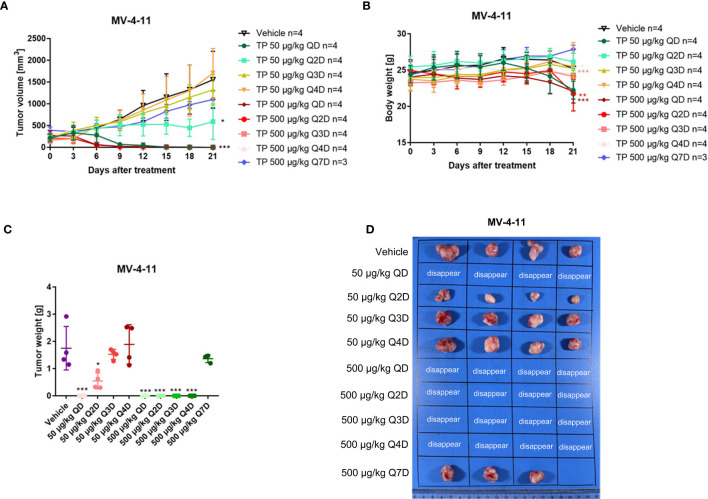
The change of the administration model for triptolide in MV-4-11 xenograft has similar anti-AML activity at the same dosage. 5×10^6^ MV-4-11 cells were injected into BALB/C nude mice subcutaneously. When the tumor volume reached 200 mm^3^, mice were randomly assigned to ten treatment groups and treated with triptolide at a dose of 50 μg/kg or 500 μg/kg daily (QD) or every other day (Q2D), every two days (Q3D), and every three days (Q4D) *via* an intraperitoneal injection for 21 consecutive days. Tumor volume **(A)** and bodyweight **(B)** was measured every two days. At the end of the study, tumors were weighed **(C)** and photographed **(D)**. *p < 0.05; **p < 0.01; ***p < 0.001.

It is known that triptolide covalently binds to a specific target (15). One of the advantages of covalent inhibitors is prolonging residence time and reducing the dosage resulting in lower adverse effects (30). However, little is known about whether the same pharmacological activity can be achieved at the same dosage following a change in the dosing cycle. Herein, we tested the anti-AML activity in MV-4-11 xenografted mice by changing the dosing cycle at the dosages of 50 μg/kg and 500 μg/kg. As is shown in **Figure 7**, all tumors disappeared when the xenograft mice were injected intraperitoneally with 500 μg/kg daily, every other day, every two days, or every three days (**Figure 7**). While there was nearly no anticancer effect for triptolide at dosages of 500 μg/kg every six days compared with other administration regimens. At the lower dosage of 50 μg/kg, daily administration of triptolide had the same pharmacological activity with a dose of 500 μg/kg daily, every other day, every two days, or every three days resulting in the disappearance of the tumor; however, the time it took for the tumor to disappear was delayed. Furthermore, the inhibitory activity of triptolide at 50 μg/kg every other day was weaker than daily administration, which was probably induced by incomplete targets blocking. A decreased antitumor effect for triptolide was observed at 50 μg/kg every two days and every three days. Moreover, most seriously body weight change in the groups at 50 μg/kg every day and 500 μg/kg every day and every other day were observed. There was slightly body weight change in the groups at 500 μg/kg every two days as well as every three days. And no body weight change in other groups decreased at the dosage of 50 μg/kg except administration every day (**Figure 7B** **)**. Thus, we have shown that a change in the dosing regimens of triptolide has similar pharmaceutical activity and reduced toxicity.

## Discussion

Natural products are a source of new chemical entities, and they play a vital role in the search for drug leads that can be optimized *via* synthetic means. In the field of oncology, the natural product triptolide is isolated from the roots of the Chinese herb *Tripterygium wilfordii* Hook f and is the herb’s primary biologically active ingredient. It possesses potent antitumor activity by regulating diverse pathways and processes in various cell types (2). The broad-spectrum antitumor activity of triptolide suggests that it targets some key molecules and events in the development of cancers. Triptolide downregulates the expression of the largest subunit of RNA polymerase (RPB1) and subsequently mediates the inhibition of cellular transcriptional levels. Unfortunately, the inhibition of transcription also results in a narrow safety window for triptolide as well as its derivatives leading to the termination of clinical trials. Meanwhile, liver and kidney injury caused by natural compound have become one of the major causes of toxicity and mortality (31, 32). Many studies and case reports have indicated that TP administration at large dose led to severe liver and renal injury (3). As a result, searching for cell lines that are sensitive to triptolide *in vitro* and *in vivo* provides a way to speed up its clinical utility. Here we demonstrated that leukemia cell lines are more sensitive to low-dose triptolide in an *in vitro* cell and an *in vivo* xenograft mouse model that was used to compare various hematological and solid tumors. Meanwhile, lower effective dosages for triptolide in anti-AML activity results in a broader safety window.

To date, few scientific papers have reported any direct targets for triptolide except XPB, TAB1, ADAM10, DCTPP1, ERα, and PC2 (29). Among them, XPB remains the most well-known and characterized target of triptolide (2). An important subunit of the TFIIH complex, XPB binds to RPB1, ensuring efficient transcription. Triptolide inhibits ATPase activity of XPB, causing degradation of RPB1 and inhibition of the transcription process (15). However, this target protein cannot fully explain its pharmacological activity induced by triptolide in various cancers. RPB1 rather than XPB was reported to contribute to the cell killing induced by triptolide in multidrug-resistant tumor cells (16). In human fibroblast cells lines, triptolide inhibited the enzymatic activity of DNA-PKcs and was predicted to bind to DNA-PKcs *via* molecular docking (33). Herein, we demonstrated that triptolide suppressed the atypical kinase activity of WSTF by decreasing the phosphorylated Y142 of H2A.X. WSTF, a transcription regulator and an atypical tyrosine protein kinase, interacts with RPB1 and is involved in the DNA repair process by phosphorylating tyrosine at the 142 residue of H2A.X when directed towards DNA damage. In contrast, the other phosphorylated residue at Ser 139 of H2A.X marks the DNA damage. The transition between H2A.X-p142 to H2A.X-p139 marks whether cells undergo a repair response or cellular apoptosis (23). We also observed that the expression of WSTF is strongly associated with triptolide’s antitumor activity. As a result, WSTF-RPB1 is necessary for triptolide to exert its antitumor activity in AML, and needs to be studied further in the near future.

Triptolide is an irreversible inhibitor through the covalent binding to its biological targets, thus suppressing their activity and inducing triptolide’s pharmacological effects. Covalent inhibitors have numerous advantages when compared to non-covalent inhibitors. Their advantage comes from covalent drugs being able to suppress the same target as a non-covalent drug using a lower dose, reducing off-target side effects. Moreover, drug resistance occurred less frequently through covalent bonding because they can overcome mutations with the binding site of their biological targets (34). In addition, covalent compounds can prolong residence time, thus further reducing the dosage (35). We observed that one high dose of triptolide every one to four days showed the same anti-AML activity within a xenograft mouse model, suggesting that an extension in administration time could achieve a similar pharmacological effect and reduces the toxic side effects at the same time. Finally, pan-genomic DNA microarray data have indicated that triptolide is a transcriptional inhibitor leading predominantly to down-regulate short-lived mRNA (36). The protein of WSTF-RPB1 has a rapid turnover rate, and functions in general transcription and its dependent DNA repair. Whether WSTF-RPB1 become a biological marker for triptolide-sensitive diseases should be studied in-depth as a new way to reduce its toxicity.

In summary, here we demonstrated that AML cell lines showed more sensitivity to triptolide *in vitro* and *in vivo*. Low-dose triptolide inhibited the proliferation of AML cell lines and induced cellular apoptosis and cell cycle arrest at G1 phase. We also monitored the toxicity of triptolide in the xenograft mice models. With increased dosing, more severe toxicity in the liver was observed. Furthermore, we observed that the expression levels of WSTF-RPB1 are positively associated with insensitivity to triptolide in hematological tumors. Triptolide downregulated WSTF-RPB1 in AML cell lines resulting in DNA damage; thus, inducing cellular apoptosis. Finally, we also observed that changing the dosing cycle for triptolide that some dosages achieved the same pharmaceutical effect with reduced toxicity. In summary, by searching for sensitive cell lines and changing the administration model, the safety window of triptolide can be increased, providing the necessary foundation for the clinical development of triptolide.

## Data Availability Statement

The datasets presented in this study can be found in online repositories. The names of the repository/repositories and accession number(s) can be found below: https://www.jianguoyun.com/p/DTsuXFsQ7OT_CRir-akE.

## Ethics Statement

The animal study was reviewed and approved by the ethics committee of the Nanjing University of Chinese Medicine.

## Author Contributions

DK and LH conceived of the presented idea. DK planed all experiments and wrote the manuscript with support from LH. LH contributed to the final manuscript. YL performed all the experiments including in **Figures 1**–**4** and **Figure S1** with the help of BF. YS finished all the experiments including in **Figures 5**–**7**. QZ did the analysis of the data and involved in discussing the mechanisms of triptolide. All authors contributed to the article and approved the submitted version.

## Funding

This study was supported by grants from the National Natural Science Foundation of China (grant: 82104270, 81873027, and 81573635), the Jiangsu Postgraduate Research & Practice Innovation Program (grant: KYCX21_1739), and the Natural Science Foundation of Nanjing University of Chinese Medicine (grant: XPT82104270).

## Conflict of Interest

The authors declare that the research was conducted in the absence of any commercial or financial relationships that could be construed as a potential conflict of interest.

## Publisher’s Note

All claims expressed in this article are solely those of the authors and do not necessarily represent those of their affiliated organizations, or those of the publisher, the editors and the reviewers. Any product that may be evaluated in this article, or claim that may be made by its manufacturer, is not guaranteed or endorsed by the publisher.
