# Supplementary figures and images for: Triptolide Shows High Sensitivity and Low Toxicity Against Acute Myeloid Leukemia Cell Lines Through Inhibiting WSTF-RNAPII Complex
Source: Front Oncol. 2022 Feb 16;12:811850. doi: 10.3389/fonc.2022.811850 (PMC8888427; doi:10.3389/fonc.2022.811850)

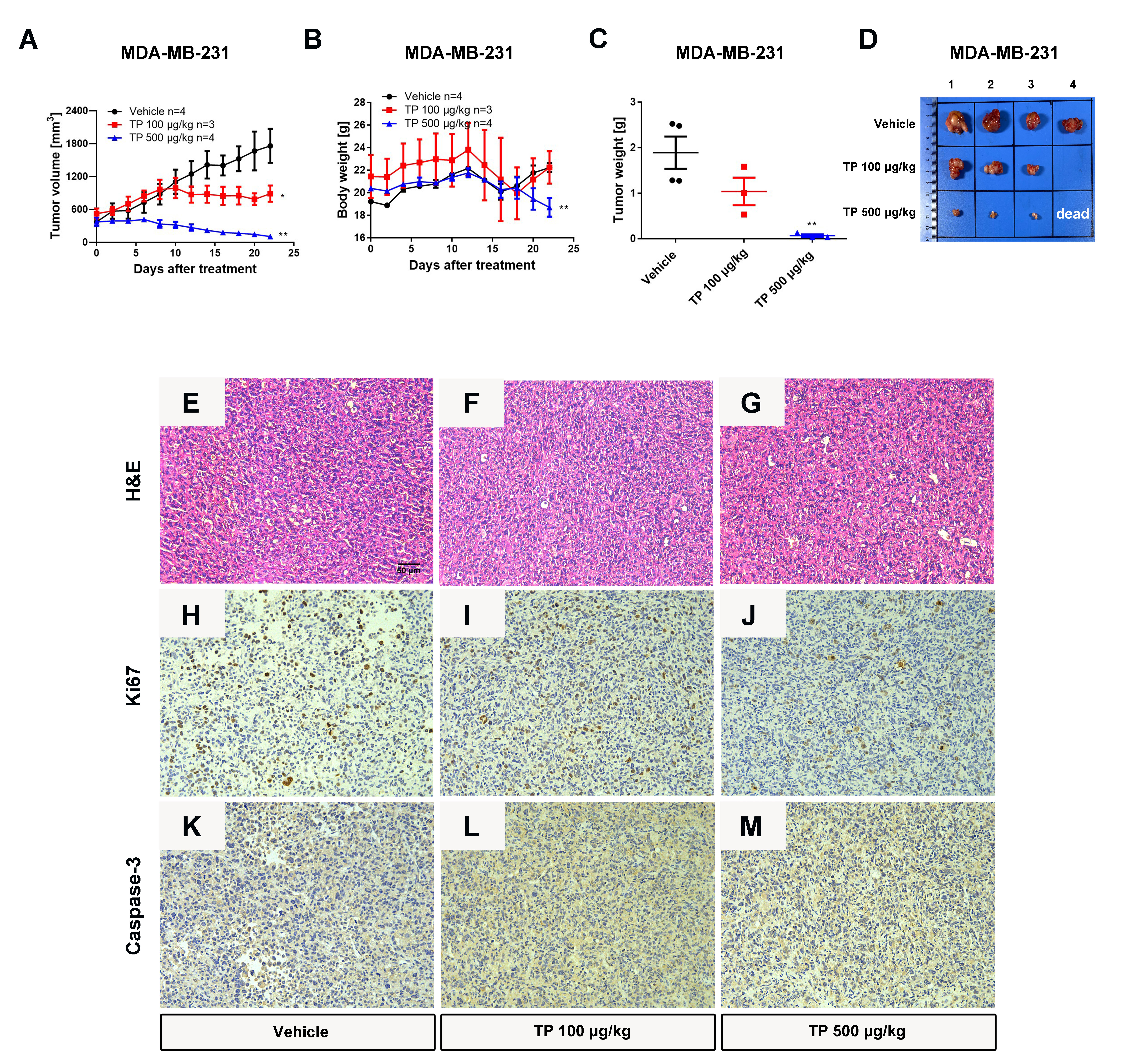

Supplement: Supplementary file 1 [file Image_1.jpg]
